# Arterioembolic Characteristics of Differentially Diluted CaHA-CMC Gels Within An Artificial Macrovascular Perfusion Model

**DOI:** 10.1093/asj/sjaf028

**Published:** 2025-02-19

**Authors:** Danny J Soares, Julia Fedorova, Yu Zhang, Akash Chandawarkar, Alexis Bowhay, Larry Blevins, Thomas J Kean, David K Funt

## Abstract

**Background:**

Despite the recently increased incidence and improved awareness of filler-induced ischemic injuries, the arterioembolic behavior of filler products has not been well described.

**Objectives:**

To evaluate the embolic behavior of varying dilutions of calcium hydroxylapatite-carboxymethylcellulose (CaHA-CMC) gel mixtures within an artificial macrovascular perfusion model of the proximal facial artery with correlation against published instances of ischemic injuries in the literature.

**Methods:**

CaHA-CMC gel mixtures were assessed through the Pulsatile Unit for the Laboratory Simulation of Arterioembolic Restrictions (PULSAR) system at different flow rates. The occlusive behavior, embolic particle size distributions, and morphological attributes were evaluated through direct photographic and videographic captures followed by digital image processing. The PubMed database was systematically queried for all published instances of CaHA-CMC-associated ischemic injuries.

**Results:**

Undiluted CaHA-CMC demonstrated highly cohesive behavior upon PULSAR inoculation, with a tendency toward proximal occlusion. Gel fragmentation resulted in a polydisperse embolic mixture averaging 0.151 ± 0.61 mm² (interquartile range: 0.006-0.022 mm²) in size. Product dilution had a profound effect on embolic behavior, with a significant reduction in average particle size (0.018 ± 0.03 mm²; interquartile range: 0.005-0.018 mm²; *P* < .0001) and complete elimination of proximally occlusive capacity for hyperdiluted mixtures compared with undiluted product (*P* = .002). Confirmed hyperdiluted CaHA-CMC-associated ischemic injuries represented only 3% of published reports, with a predominantly self-limited clinical course.

**Conclusions:**

Embolized CaHA-CMC gels produce polydisperse particle mixtures with a preponderance of microparticles. Hyperdilution profoundly reduced the proximally occlusive potential of the product.

The recent rise in the incidence of arterioembolic ischemic injuries stemming from cosmetic dermal filler injections has motivated new efforts to elucidate the behavior of viscoelastic colloidal mixtures within arterial environments.^1-3^ Given their shear-thinning nature, this class of hydrogel products exhibits a nonlinear increase in fluidity when exposed to elevated shear strain rates, leading to complex fragmentation and occlusion dynamics that are difficult to predict.^4^ Consequently, the study of filler embolization events has benefited from experimental models featuring simulated arterial environments that enable the direct observation of gel mixtures in variable-flow tubular networks. Recently, the newly validated Pulsatile Unit for the Laboratory Simulation of Arterioembolic Restrictions (PULSAR) system has helped to elucidate the mechanical behavior of hyaluronic acid (HA) fillers within a macrovascular (>1 mm) proximal facial artery model.^5^ Specifically, such studies have shown that HA gels can undergo extensive fragmentation into microparticles, evoking distal microembolic showers, and microcirculatory obstruction, with the possibility of proximal plug formation at sufficiently high embolic burdens or at physiologically low flow rates. In addition, the size, shape, and occlusive potential of HA filler microparticles are heavily influenced by the material's viscoelastic profile, with the gel's loss factor (tan *δ*)—an indicator of the relative viscous-over-elastic dominance in a gel—serving as a predictor of embolic particle morphology.^5^

Despite the widespread clinical usage of HA products in traditional aesthetic medicine applications, non-HA filler materials, such as injectable calcium hydroxylapatite-carboxymethylcellulose (CaHA-CMC), continue to be clinically employed because of their favorable biostimulatory effects. In the United States, CaHA-CMC injectable gel (Radiesse, Merz Aesthetics, Raleigh, NC) is an FDA-approved dermal filler consisting of CaHA microspheres (25-40 µm in diameter, 30% w/v) suspended in a CMC gel carrier (70%). Recently, this formulation has gained popularity in hyperdilute aqueous preparations for bioregenerative treatments, demonstrating significant improvements in skin quality and elasticity.^6-10^ The effects of titrated aqueous dilutions of CaHA-CMC products have been recently shown to significantly impact product rheology, leading to the attenuation of material cohesion and elastic strength.^11,12^ Accordingly, the occlusive potential of hyperdiluted CaHA-CMC mixtures (ie, dilutions >1:1, by volume) has also been hypothesized to be lessened relative to hypodiluted (ie, <1:1) or undiluted products. In this study, we evaluate the embolic behavior of 4 differentially diluted CaHA-CMC gel mixtures within a macrovascular facial artery model and correlate the embolic particle morphology and occlusive potential to clinical reports of ischemic adverse events in the published literature.

## METHODS

### Simulated Macrocirculatory System Design and Calibration

**Figure 1.**
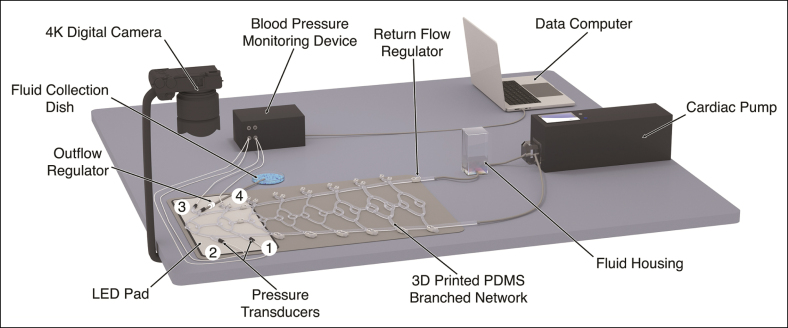
Components of the experimental setup for the PULSAR system. A 3-dimensionally printed, dichotomously branched tubular network composed of polydimethylsiloxane and obeying Murray's law of minimum work is coupled to a modified cardiac pump. The distal portion of the tubular network is designed to simulate the diameters of the proximal paramandibular facial artery and its main branches, with a photographic and videographic capturing system positioned directly over the circuit for real time monitoring of embolic dissemination of inoculated products. Pressure transducers, positioned at multiple points, monitor the pulsatile waveform to ensure a physiologically matched pressure profile. This figure originally appeared within Soares and McCarthy,^5^ published by MDPI (Basel, Switzerland) under a Creative Commons Attribution (CC BY) license agreement, which permits reproduction of the image with proper attribution to the original work. PULSAR, Pulsatile Unit for the Laboratory Simulation of Arterioembolic Restrictions.

**Figure 2.**
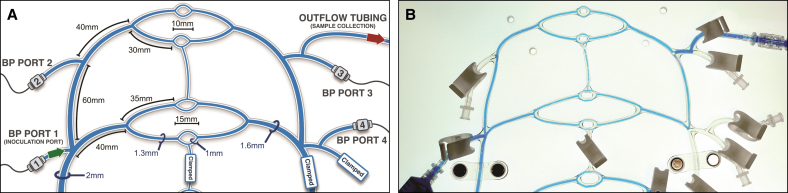
The distal PULSAR network, designed to simulate the proximal facial artery and its main branches. (A) Diagram of the tubular system with dimensional parameters of each branch. Blood pressure transducers are installed at each of the 4 access points, with the inoculation port (green arrow) providing inoculation access and the collection port enabling sampling of returning fluid (arrow). (B) Photograph of the distal PULSAR network and its access ports, containing intraluminal dyed fluid for contrast. This figure originally appeared within Soares and McCarthy,^5^ published by MDPI under a Creative Commons Attribution (CC BY) license agreement, which permits reproduction of the image with proper attribution to the original work. PULSAR, Pulsatile Unit for the Laboratory Simulation of Arterioembolic Restrictions.

**Figure 3.**
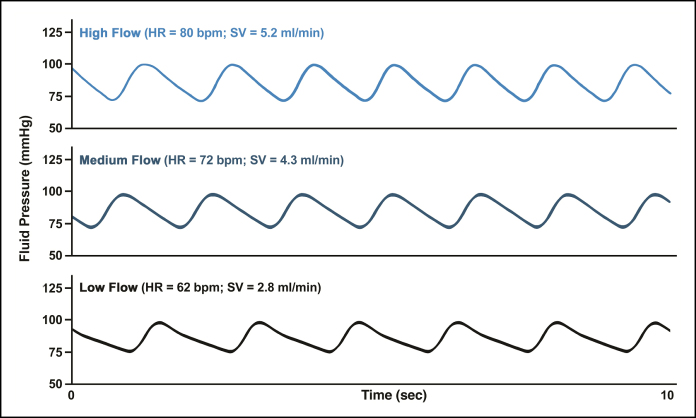
Sample PULSAR waveforms for the inoculation access port during system operation. The pulsatile waveform remained within physiological blood pressure ranges reported for medium arteries in humans, with relative uniformity of pulse pressure differential at all flow rates. HR, heart rate; PULSAR, Pulsatile Unit for the Laboratory Simulation of Arterioembolic Restrictions; SV, stroke volume.

A simulated macrovascular environment of the paramandibular facial artery and its proximal branches was assembled according to the previously published PULSAR protocols.^5^ The PULSAR system features a 3-dimensionally printed, dichotomously branched tubular circuit composed of a Shore A-44 polydimethylsiloxane elastomer obeying Murray's law (principle governing optimal branching in vascular systems, where *R*_p_^_3_^=*R*_d1_^_3_^+…+*R*_dn_^_3_^, and *R*_p_ and *R*_d1_/*R*_d2_/*R*_d*n*_ are the radii of the parent and daughter branches, respectively), coupled to a modified cardiac pump (EDU-Heart Pump, Trandomed, Ningbo, China) and terminal branch pressure transducers, as displayed in Figure 1.^13^ The distal portion of the circuit employed in this study is comprised of 3 generations of bifurcated branching points spanning luminal diameters of 2.0, 1.6, 1.3, and 1.0 mm, consistent with reported ranges for proximal facial artery and its branches in humans, equipped with a proximal inoculation port and a distal fluid collection port (Figure 2).^14-16^ The circulating fluid consists of a 50% aqueous glycerol mixture, by volume, composed of high-purity glycerol (G7893, Sigma-Aldrich, Inc., St Louis, MO) and deionized water at 20 °C, approximating the viscosity of human blood.^17^ Before system activation, the circuit is fully primed with the glycerol mixture, and all air pockets are cleared. The pump is subsequently initiated, and its stroke volume and rate settings are adjusted to achieve a pulsatile waveform within the physiological pressure (90-100/70-80 mm Hg) and flow rate (7.3-35.3 mL/min) ranges reported for the proximal facial artery in humans (Figure 3).^18-22^ For this study, calibration parameters for low, medium, and high flow rate settings are displayed in Table 1.

**Table 1.** Calibrated PULSAR Settings for Different Physiological Facial Artery Flow Rates

Parameter	Low flow (9.6 mL/min)	Medium flow (18.6 mL/min)	High flow (36.0 mL/min)
Stroke rate (strokes/min)	62	72	80
Stroke volume (mL/stroke)	2.8	4.3	5.2
Pump output (mL/min)	173.6	309.6	416.0
Average systolic pressure (mm Hg)	97.97 ± 3.08	96.99 ± 1.98	102.12 ± 1.84
Average diastolic pressure (mm Hg)	73.14 ± 0.15	68.03 ± 1.23	69.40 ± 0.11
Average mean arterial pressure (mm Hg)	81.42 ± 0.31	77.68 ± 0.16	80.31 ± 0.07

PULSAR, Pulsatile Unit for the Laboratory Simulation of Arterioembolic Restrictions.

### System Inoculation and Videographic Capture of Embolic Dissemination

Experimental testing was conducted between July and October 2024. Product mixtures were prepared immediately before injection into the system, consisting of undiluted and 1:0.5, 1:1, and 1:2 product dilutions. For dilute mixtures, 1.5 mL CaHA-CMC filler (Radiesse, Merz Aesthetics) was combined with 0.75, 1.5, and 3 mL of normal saline (0.9% sodium chloride USP; Hospira, Lake Forest, IL) yielding the 1:0.5, 1:1, and 1:2 dilutions, respectively. The product and saline components were placed into separate 3 mL Luer lock syringes and connected through a male-to-male attachment (Rapidfill Connector, Baxter Healthcare, Deerfield, IL) and subsequently homogenized through back-and-forth mixing 20 times. Reported rheological parameters for diluted and undiluted CaHA-CMC mixtures are presented in Table 2, derived from published data by McCarthy et al, and the meaning of key rheological parameters is summarized in Supplemental Table 1.^12^ Immediately upon mixing, 0.2 mL of the product was drawn into a 1 mL syringe for immediate injection into the PULSAR system. System inoculation was performed by injection of a 0.2 mL bolus of the CaHA-CMC mixture through a 27 G, 50 mm microcannula (Steriglide, TSK Laboratories, Vancouver, BC) at a manual injection rate of ∼0.01 mL/s. Videographic capture of filler dispersal was achieved through a stationary high-resolution recording system employing a 24.2 megapixel digital single-lens reflex (DSLR) camera (Alpha 6600, E18-135 mm Lens, Sony, New York, NY) over 1 min and until all of the visible inoculum dissipated from the system or resulted in terminal, static flow restriction. Upon completion of each inoculation session, the system was cleared of any gel plugs and any filler-contaminated glycerol solution was discarded.

**Table 2.** Rheological Parameters of CaHA-CMC Mixtures Employed in this Study.^12^

CaHA-CMC mixture	Elastic modulus (*G*′) [Pa (0.1 Hz)]	Viscous modulus (*G*″) [Pa (0.1 Hz)]	Complex modulus (G*) [Pa (0.1 Hz)]	Loss factor Tan (*δ*) (0.1 Hz)
Undiluted	962.00	422.90	1050.85	0.44
1:0.5	40.78	42.61	58.98	1.05
1:1	8.37	12.42	14.98	1.48
1:2	1.02	2.51	2.71	2.46

CaHA-CMC, calcium hydroxylapatite-carboxymethylcellulose.

### Gel Particle Size and Morphological Analysis

Macroscopic particle size analysis was performed through digital image assessments of high-resolution photographs of returning circulating fluid collected immediately following inoculation. Embolized fluid was diverted onto a 100 × 20 mm Petri dish (Pyrex Dish, Corning, Rosemont, IL) coupled with a 0.01 mm glass calibrating ruler (Microscope Calibration Slide, MUHWA Scientific, Zhanjiang, CN). Still photographs were captured through a 4 K, 20.9-megapixel DSLR camera (D7500, Nikon, Melville, NY) equipped with a macro lens (Nikkor 105 mm f/2.8 G, Nikon) with the background lit through an LED pad (LitEnergy A4 Ultra-thin LED Lightbox, GGE Corp., Shenzhen, CN). High-resolution images were assessed based on previously reported protocols.^5,23^ Gel particles were analyzed using ImageJ version 1.5.4 (ImageJ, National Institutes of Health, Bethesda, MD). Since the CaHA is solid and white, this process forewent the color deconvolution steps in our previous study and immediately converted the image to an 8-bit binary image. Next, the image size was calibrated based on the calibration slide glass. After calibration, a standard image threshold was applied and adjusted to highlight the particles and particle aggregates. A region of interest was defined, and nonparticulate artifacts were cleared from the image. Particle analysis encompassed area (mm^2^), major- and minor-axis lengths (mm), perimeter (mm), circularity, roundness, aspect ratios, and solidity (please see Supplemental Table 1 for definitions of these attributes). The interquartile range (IQR) for particle area was also derived, defined as the range encompassing the middle 50% of the particle size dataset between the 25th and 75th percentiles.

### CaHA Filler-Associated Ischemic Injury Literature Review

A systematic review of the published English literature was conducted to identify vascular complications associated with CaHA-CMC fillers, with a focus on evaluating complication rates relative to the presence of product hyperdilution. PubMed was queried for case reports and research articles published up to October 2024 employing the following keywords: (“calcium hydroxylapatite” OR “Radiesse” OR “CaHA” OR “Calcium Hydroxylapatite Filler”) AND (“ischemia” OR “occlusion” OR “blindness” OR “vascular” OR “vision loss” OR “stroke” OR “skin necrosis”). Medical Subject Headings terms were applied to refine the search. Additional case reports not captured in the primary search were identified through a backward citation analysis of references in relevant articles. Only descriptive manuscripts documenting vascular complications related to CaHA fillers in humans were included. Reports were classified as involving either hyperdiluted (≥1:1) or nonhyperdiluted (undiluted or <1:1) products, determined from the described dilution ratio or inferred from the clinical application for nonhyperdiluted (eg, nasal dorsal augmentation, chin augmentation, nasolabial-fold effacement, and glabellar-fold effacement) and hyperdiluted indications (eg, cheek volumization).

### Statistical Analysis

Statistical analysis was carried out using GraphPad Prism (Version 10.3.0, GraphPad Software, LLC, San Diego, CA). One-way analysis of variance and Kruskal–Wallis *H* tests were used to assess statistical significance between and within dilution and flow rate groups for parametric and nonparametric data, respectively. Fisher's exact test was employed to evaluate the statistical significance for nominal data, such as the presence of system obstruction with hyperdiluted and nonhyperdiluted product mixtures. Graphs and illustrations were made using GraphPad Prism and Adobe Photoshop (Adobe Systems, San Jose, CA).

## RESULTS

### Embolic Particle Attributes and Occlusive Dynamics Outcomes

**Figure 4.**
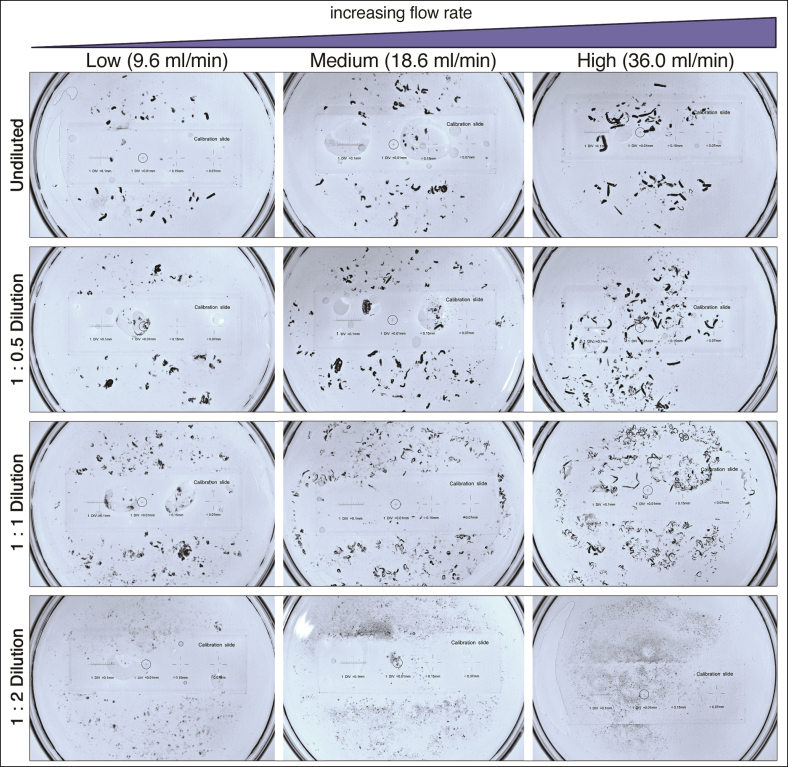
Appearance of embolic fragments of undiluted and 1:0.5, 1:1, and 1:2 dilute CaHA-CMC mixtures following inoculation into the PULSAR system at low, medium, and high flow rates. Particle size progressively decreased with increasing dilution ratio and demonstrated a tendency toward more elongated fragment morphology with increased flow rates. CaHA-CMC, calcium hydroxylapatite-carboxymethylcellulose; PULSAR, Pulsatile Unit for the Laboratory Simulation of Arterioembolic Restrictions.

Particle size and morphological attributes for all product mixtures and flow rates are visually displayed in Figure 4, with specific measurement data summarized in Supplemental Tables 2 and 3. Undiluted CaHA-CMC boluses initially remained cohesive but subsequently dispersed upon flowing past branching points. Upon system dissemination, the fragmentation of undiluted product generated a polydisperse embolic mixture with an average particle size of 0.151 ± 0.61 mm² (range: 0.004-11.189 mm²; IQR: 0.006-0.022 mm²; mode: 0.004 mm²), average major axis of 310 ± 588 µm (IQR: 105-217 µm, mode: 76 µm), and average minor axis of 183 ± 257 µm (IQR: 72-121 µm; mode: 62 µm) for aggregated data inclusive of all flow rates. Particle size increased with flow rate, ranging from 0.104 ± 0.455 mm² at low flow to 0.174 ± 0.607 mm² at medium flow and 0.174 ± 0.759 mm² at high flow (*P* < .0001). This increase in size was primarily driven by particle elongation, as evidenced by the corresponding rise in major axis length.

**Figure 5.**
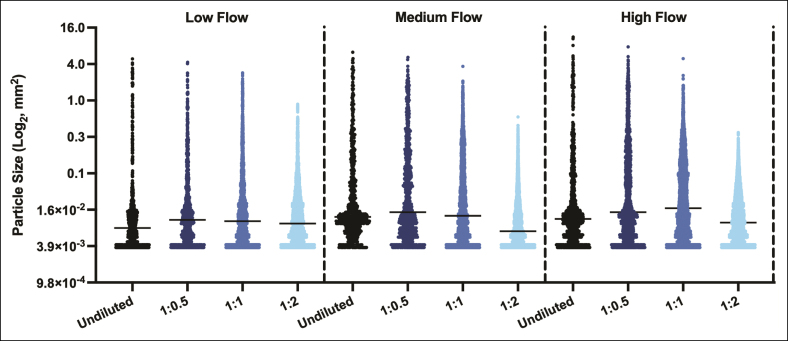
Particle size distributions for undiluted and 1:0.5, 1:1, and 1:2 diluted CaHA-CMC mixtures. Product hyperdilution (1:1, 1:2) resulted in a shift toward increased prevalence of microparticles compared with the undiluted or hypodiluted (1:0.5) mixtures at all flow rates. CaHA-CMC, calcium hydroxylapatite-carboxymethylcellulose.

In contrast, a significant reduction in particle size was observed with increasing dilution, as displayed in Figure 5. Specifically, the average particle size decreased from 0.151 ± 0.61 mm² in the undiluted product to 0.149 ± 0.46 mm² at 1:0.5 dilution, 0.065 ± 0.18 mm² at 1:1 dilution, and 0.018 ± 0.03 mm² at 1:2 dilution (*P* < .0001). Dimensional analysis of the embolic particles showed a progressive decrease in major and minor axes as dilution increased, with values ranging from 310 ± 568 µm (major axis) to 185 ± 257 µm (minor axis) in the undiluted state vs 170 ± 99 µm (major axis) to 103 ± 68 µm (minor axis) at a 1:2 dilution (*P* < .0001). Morphologically, dispersed fragments exhibited a predominantly ovoid shape across all mixtures, with an average aspect ratio of 1.69 ± 0.76. Notably, hypodiluted mixtures also displayed a tendency toward elongation with increased flow rate, although 1:2 hyperdilutions demonstrated marked disintegration into smaller microparticles at increasing flow rate settings, consistent with its reduced cohesion.

**Figure 6.**
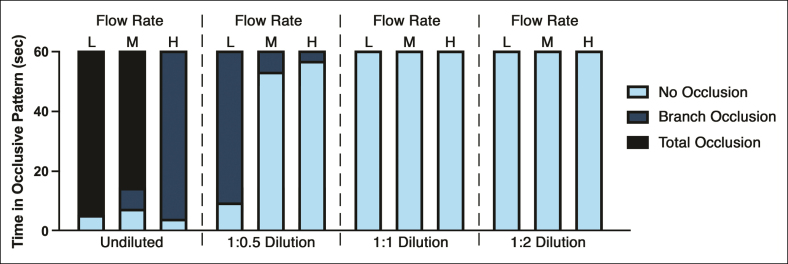
Duration of specific occlusive patterns (ie, no occlusion, branch occlusion, and total system occlusion) observed for each CaHA-CMC mixture during the first minute following PULSAR inoculation. The undiluted product exhibited a significantly higher propensity for persistent total system occlusion compared to the dilute varieties. Hyperdilution at ratios of 1:1 and 1:2 completely eliminated the product's macrovascular obstructive potential. CaHA-CMC, calcium hydroxylapatite-carboxymethylcellulose; H, high flow; L, low flow; M, medium flow; PULSAR, Pulsatile Unit for the Laboratory Simulation of Arterioembolic Restrictions.

The macro-occlusive potential of each CaHA-CMC mixture is summarized in Figure 6, with sample inoculation footage for the nondiluted and 1:2 hyperdiluted products shown in Videos 1 and 2, respectively. The nondiluted product showed the greatest propensity for total system occlusion, particularly at low and medium flow rates, resulting in sustained obstruction. However, at higher flow rates, the occlusive potential shifted toward a predominance of branch occlusion rather than total system blockage, consistent with the greater fluid momentum at high flow. Dilution markedly altered the proximal occlusive capabilities of the product, with the 1:1 and 1:2 formulations failing to induce any type of occlusion at all flow rates compared with hypo/nondiluted mixtures (*P* = .002). Furthermore, dilution also impacted the product's tendency to leave residual coating along the tubular wall, with the 1:1 and 1:2 formulations failing to generate any macroscopic proximal residue.

### Literature Review Findings

A total of 54 potential publications were identified through the initial PubMed search, of which 30 manuscripts met the inclusion criteria for unique case reports of suspected ischemic injuries associated with CaHA-CMC, summarized in Supplemental Table 4.^24-53^ Collectively, the assessed literature comprised a total of 37 individual patient cases, with 19 instances of isolated facial skin ischemia (51.4%), 16 reports of visual deficits with or without skin ischemia (43.2%), and 1 instance each of pulmonary embolism and trigeminal neuropathy. The majority of ischemic complications resulted from nasal dorsal augmentation (35.1%) and nasolabial-fold treatments (27.1%), with others arising from injections into the cheek (16.2%), glabella (8.1%), chin (5.4%), temple (5.4%), and vocal cords (2.7%). In all, only one reported instance (2.7%) involved the confirmed use of a hyperdiluted CaHA-CMC mixture (1:2), injected into the cheek, resulting in a self-limited occurrence of livedoid skin changes without progression to tissue necrosis.^53^

## DISCUSSION

The viscoelastic properties of dermal fillers, stemming from their colloidal structure, combine the attributes of solid-like rigidity with liquid-like fluidity, which vary with the magnitude and rate of the applied strain.^4^ Clinically, this hybrid behavior has been harnessed to create products that can resist deformation at low shear strains but readily undergo cannulated extrusion in high shear, permitting injection through fine needles. The enhanced fluidity of hydrogels under high shear, a property known as shear-thinning, occurs because of topological alterations in the material's structure, in which entangled polymer chains gradually uncoil, disentangle, or break, permitting chain alignment and the onset of plastic flow (Supplemental Figure 1).^54^ Resultantly, the behavior of dermal fillers within the arteriovascular system represents a complex and dynamic process that is highly dependent on material and vascular parameters and is therefore challenging to predict.^55^ Nonetheless, the fragmentation and dispersal of gel emboli within medium-to-small arteries remain arguably the most important product-specific determinant of the ultimate perfusion deficit within a vascular network and, subsequently, the likelihood of ischemic injury.^1,56^ Already, recent insights obtained from studies in the rat inferior epigastric model indicate a substantially greater risk of tissue necrosis with chemically crosslinked HA products than with noncrosslinked HA gels.^2,3^ This reduced degree of ischemic risk likely stems from the decreased gel strength, elasticity, and cohesion of noncrosslinked HA mixtures, characteristics that favor the distal dispersal of the filler and facilitate the mechanical and enzymatic clearance of embolized material in the distal microcirculation.^55^

Despite these advances in our understanding of the behavior of HA gel emboli within vascular environments, the embolic characteristics of non-HA fillers, particularly CaHA-CMC composites, have remained largely unexplored. Structurally, CaHA-CMC products exhibit significant differences in chemical composition and mechanical attributes compared to HA gels, which are reflected in the product's rheological parameters. Specifically, their biphasic constitution, consisting of CaHA microspheres dispersed in an entangled solution of noncrosslinked CMC polymer chains, confers these gels added strength yet greater ductility, reducing their propensity for brittle failure. Resultantly, CaHA-CMC gels extrude at higher forces than most HA products but demonstrate a “stringy” quality, suggestive of a high degree of cohesion despite its composite nature.^57^ This quality is evident in the product's behavior upon inoculation into the PULSAR system, where it displayed significant functional cohesion, akin to other cohesive HA gels, resisting fragmentation until sheared past branching points.

Our findings confirm that CaHA-CMC composites, like HA gels, undergo significant fragmentation in a branched macrovascular model, generating a polydisperse mixture of microparticles averaging 310 µm in major axis but spanning a broad range of sizes, from ∼30 µm (individual microspheres) up to ∼6 mm. The low average fragment sizes (0.151 mm^2^, mode: 0.004 mm²) of undiluted CaHA-CMC indicate a preponderance of microparticles, akin to other elastically strong HA gels, which is likely further favored by the product's microparticulate nature.^5^ Nonetheless, the product's tendency to generate elongated fragments with increasing flow rates showcases a behavior more typical of softer HA gels, in line with the gel's relatively high tan *δ*, despite its robust strength.^12^ These product attributes—namely, elevated gel strength, plasticity, and cohesion—explain the obstructive characteristics observed with the embolized CaHA-CMC product. In this experiment, undiluted CaHA-CMC revealed a tendency for proximal macrovascular occlusion, akin to but beyond that of other high elastic strength HA gels—as observed in our previous PULSAR experiment—with the product causing branch occlusion even at high flow rates and showing a predisposition for adhesive, residual coating of tubular walls.^5^ Furthermore, proximally lodged fragments also served as reservoirs for distally disseminating microemboli, mirroring the behavior observed with HA fillers. Clinically, this suggests a complex and dynamic obstructive pattern in product-related vascular injuries, with a semi-random distribution of occlusive proximal and distal emboli within a perfusing network.^5^

Aqueous dilution profoundly altered the embolic and occlusive behavior of CaHA-CMC gels. Previously, the dilutional modification of CaHA-CMC products was shown to significantly diminish material cohesion—the gel's capacity to resist fragmentation—and gel stiffness, defined as its ability to resist deformation.^11,12^ This attenuation of core mechanical properties, coupled with increased fluidity, has been theorized to reduce particle size and lower the likelihood of proximal vascular occlusion.^11^ Our results corroborate these hypotheses, showing that progressive dilution of CaHA-CMC significantly reduces particle size through dispersion, ranging from an undiluted average of 0.151 mm² down to 0.019 mm² in hyperdiluted formulations. Moreover, the ability of CaHA-CMC gels to elicit proximal, macrovascular occlusion was significantly reduced even at low-volume dilutions of 1:0.5, being virtually nonexistent at 1:1 and 1:2 dilution ratios.

**Figure 7.**
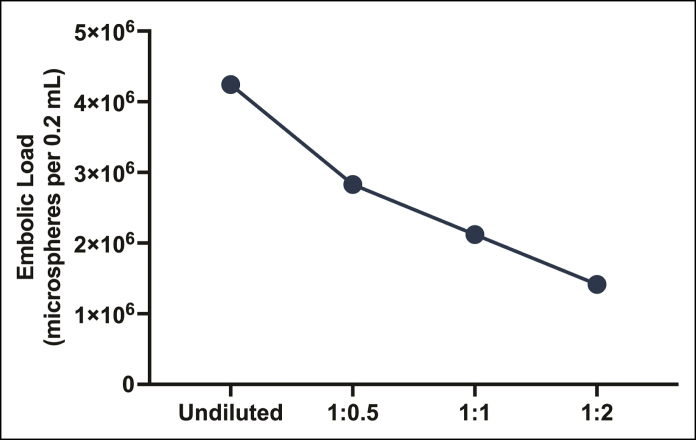
Relationship between product dilution and number of potential embolic microparticles. The number of occlusive microparticulate contents per unit volume decreases with increasing aqueous dilutional volume, thereby reducing the potential embolic burden of inoculated hyperdilute mixtures.

Collectively, these findings highlight the limited potential of diluted CaHA-CMC gel inoculations for causing proximal macrovascular occlusion, suggesting a more distal, microvascular embolic target. Given that the total cross-sectional area of the distal circulation increases exponentially with branching (a natural consequence of Murray's law), and that the relative embolic content of a mixture decreases with aqueous dilution (Figure 7), it is likely that the risk of ischemic skin hypoperfusion is significantly lowered through product hyperdilution.^55^ This postulate is supported by our survey of the published literature, which reveals that the vast majority of published instances of CaHA-CMC ischemic injuries (97%) occur with non/hypodiluted mixtures, and a recent analysis of the FDA's Manufacturer and User Facility Device Experience database showing similar findings.^11^ Although these analyses are prone to sampling bias due to a higher usage of nonhyperdiluted products, the decreased risk of ischemic skin injury has also been confirmed for diluted CaHA-CMC gels and high-fluidity, biphasic polymethylmethacrylate-collagen gels in rabbit ear model experiments.^58,59^ Nonetheless, because of their reduced particle size, hyperdiluted mixtures still pose a risk of distant dissemination through distal microanastomoses, potentially affecting ischemia-sensitive tissues like the retina and brain, especially with large-volume inoculations.^55^

Although this experimental evaluation provides valuable insights into the mechanical properties of CaHA-CMC products within a branched macrovascular perfusion model, several limitations warrant consideration. First, the in vitro design inherently excludes critical physiological factors known to be involved in the embolization process, such as the coagulation cascade, reflexive vasomotor responses, and bioenzymatic/inflammatory processes intrinsic to circulating whole blood in living arteries. These factors may significantly influence the behavior and occlusive potential of embolized materials in vivo, limiting our ability to directly extrapolate into the clinical presentation of these injuries. Secondly, the absence of a microvascular network restricts our ability to evaluate the distal dissemination and ultimate occlusive endpoints of microparticles. Additionally, the use of a fixed bolus size, uniform injection rate, and single injection device limits our assessment of technical aspects that, in reality, are characterized by significant variability in clinical practice. Future investigations will benefit from the use of microvascular networks, a range of injection devices and volumes, and a physiological circulating fluid/biological arterial network for the comprehensive study of filler embolization dynamics relevant to clinical practice.

## CONCLUSIONS

This study provides novel insights into the embolic and occlusive behavior of CaHA-CMC gels within a branched macrovascular perfusion model and the influence of dilution on embolic particle dynamics and occlusive potential. Undiluted CaHA-CMC demonstrated highly cohesive behavior upon system inoculation, with dissemination producing a polydisperse embolic mixture, and an overall propensity for proximal macrovascular occlusion. In contrast, hyperdilution profoundly reduced the apparent cohesion, particle size, and proximal occlusive potential of the product. Hyperdiluted mixtures were associated with a predominantly distal dissemination pattern, consistent with findings in the literature suggesting a significantly lower incidence of ischemic complications with diluted formulations. Although these findings underscore the potential clinical benefits of hyperdilution in minimizing the risk of proximal vascular occlusion, they also highlight the continued need for caution because of the potential for distant dissemination and microvascular injury, particularly in ischemia-sensitive tissues. Future studies incorporating physiological variables, microvascular networks, and diverse injection parameters will be essential to further enhance the translational relevance of these findings and to optimize safety in clinical practice.

## Supplemental Material

This article contains supplemental material located online at https://doi.org/10.1093/asj/sjaf028.

## Supplementary Material

sjaf028_Supplementary_Data
