# Supplementary material for: Arterioembolic Characteristics of Differentially Diluted CaHA-CMC Gels Within An Artificial Macrovascular Perfusion Model
Source: Aesthet Surg J. 2025 Feb 19;45(6):645–53. doi: 10.1093/asj/sjaf028 (PMC12209786; doi:10.1093/asj/sjaf028)
Supplement: sjaf028_Supplementary_Data [file sjaf028_Supplementary_Data.zip › Table S1.docx]

**Table S1. Summary description of key rheological parameters and particle morphology attributes.**

| Parameter | Units | Description |
| --- | --- | --- |
| Rheometry | | |
| Elastic Modulus (G') | Pa | Parameter describing the solid-like component of viscoelastic materialbehavior, quantified as the ability to elastically store energy or resist permanent deformation. |
| Viscous Modulus (G") | Pa | Parameter describing the fluid-like component of viscoelastic material behavior, quantified as the ability to viscously dissipate energy or resist changes in the rate of deformation or flow. |
| Complex Modulus (G*) | Pa | Complex number quantifying the overall strength of a viscoelastic material or its ability to resist deformation. The G* modulus combines the magnitude of G' (real or in-phase component) and G" (imaginary or out-phase component. The G* is represented by the slope of the curve on a shear stress vs shear strain plot. |
| Loss Factor (tan δ) | -- | Also known as the dissipation factor, it represents the relative magnitude of a material's viscous strength to its elastic strength, calculated as the ratio G"/G' or the tangent of the phase angle δ. |
| Particle Morphology | | |
| Circularity | -- | A measure of how closely a particle's shape resembles a perfect smooth circle. A value of 1 indicates a perfect smooth circle, with lower values representing more irregular or elongated shapes. |
| Aspect Ratio | -- | The ratio of the particle's major axis to its minor axis, describing its elongation. Higher values indicate more elongated shapes, while a value of 1 represents a perfect sphere or circle. |
| Roundness | -- | A measure of a particle’s similarity to a perfect circle, regardless of edge smoothness. Values range from 0 to 1, with 1 indicating a perfect circle. Lower values reflect elongation. |
| Solidity | -- | The ratio of the particle's actual area to the area of its convex hull (the smallest convex shape enclosing the particle). A value close to 1 indicates minimal concavities, while lower values reflect increased surface irregularity or concavity. |
