# Supplementary material for: Arterioembolic Characteristics of Differentially Diluted CaHA-CMC Gels Within An Artificial Macrovascular Perfusion Model
Source: Aesthet Surg J. 2025 Feb 19;45(6):645–53. doi: 10.1093/asj/sjaf028 (PMC12209786; doi:10.1093/asj/sjaf028)
Supplement: sjaf028_Supplementary_Data [file sjaf028_Supplementary_Data.zip › Table S2.docx]

**Table S2. Particle size measurements for each mixture at different flow rates.**

| Mixture | | Area (mm^2) | | | Perimeter (mm) | | | Major (mm) | | | Minor (mm) | | |
| --- | --- | --- | --- | --- | --- | --- | --- | --- | --- | --- | --- | --- | --- |
|  |  | Low Flow | Medium Flow | High Flow | Low Flow | Medium Flow | High Flow | Low Flow | Medium Flow | High Flow | Low Flow | Medium Flow | High Flow |
| Undiluted | Mean | 0.104 | 0.174 | 0.174 | 0.876 | 1.235 | 1.093 | 0.247 | 0.342 | 0.340 | 0.145 | 0.218 | 0.191 |
|  | SD | 0.455 | 0.607 | 0.759 | 1.785 | 2.130 | 2.313 | 0.472 | 0.538 | 0.693 | 0.221 | 0.294 | 0.256 |
|  | Min | 0.004 | 0.004 | 0.004 | 0.194 | 0.194 | 0.194 | 0.070 | 0.073 | 0.073 | 0.034 | 0.035 | 0.031 |
|  | Max | 4.869 | 6.262 | 11.189 | 12.898 | 18.488 | 32.716 | 4.666 | 4.120 | 6.666 | 1.601 | 2.549 | 2.565 |
|  | 75th Percentile | 0.015 | 0.048 | 0.032 | 0.439 | 1.136 | 0.682 | 0.138 | 0.276 | 0.237 | 0.136 | 0.220 | 0.173 |
|  | 25th Percentile | 0.005 | 0.008 | 0.006 | 0.298 | 0.376 | 0.268 | 0.133 | 0.126 | 0.091 | 0.044 | 0.084 | 0.090 |
| 1 to 0.5 | Mean | 0.082 | 0.173 | 0.174 | 0.896 | 1.319 | 1.213 | 0.248 | 0.391 | 0.391 | 0.161 | 0.232 | 0.230 |
|  | SD | 0.319 | 0.469 | 0.513 | 1.508 | 1.837 | 1.774 | 0.340 | 0.526 | 0.564 | 0.202 | 0.268 | 0.260 |
|  | Min | 0.004 | 0.004 | 0.004 | 0.194 | 0.194 | 0.194 | 0.070 | 0.072 | 0.070 | 0.034 | 0.039 | 0.024 |
|  | Max | 4.283 | 5.184 | 7.693 | 16.819 | 19.266 | 23.710 | 3.202 | 4.398 | 5.994 | 2.010 | 1.604 | 2.666 |
|  | 75th Percentile | 0.025 | 0.093 | 0.093 | 0.573 | 1.656 | 1.383 | 0.184 | 0.612 | 0.439 | 0.172 | 0.194 | 0.271 |
|  | 25th Percentile | 0.006 | 0.007 | 0.007 | 0.255 | 0.316 | 0.353 | 0.089 | 0.112 | 0.133 | 0.079 | 0.078 | 0.066 |
| 1 to 1 | Mean | 0.057 | 0.071 | 0.066 | 0.855 | 0.997 | 0.914 | 0.230 | 0.281 | 0.291 | 0.143 | 0.167 | 0.169 |
|  | SD | 0.200 | 0.182 | 0.160 | 1.250 | 1.134 | 0.942 | 0.261 | 0.292 | 0.284 | 0.165 | 0.165 | 0.140 |
|  | Min | 0.004 | 0.004 | 0.004 | 0.194 | 0.194 | 0.194 | 0.072 | 0.069 | 0.070 | 0.032 | 0.029 | 0.032 |
|  | Max | 2.858 | 3.660 | 4.935 | 14.867 | 12.094 | 16.145 | 3.249 | 3.208 | 3.693 | 1.457 | 1.660 | 2.194 |
|  | 75th Percentile | 0.024 | 0.051 | 0.062 | 0.694 | 1.424 | 1.674 | 0.229 | 0.340 | 0.439 | 0.135 | 0.191 | 0.179 |
|  | 25th Percentile | 0.005 | 0.006 | 0.007 | 0.237 | 0.353 | 0.285 | 0.094 | 0.137 | 0.107 | 0.069 | 0.051 | 0.082 |
| 1 to 2 | Mean | 0.023 | 0.016 | 0.017 | 0.626 | 0.525 | 0.561 | 0.178 | 0.160 | 0.172 | 0.112 | 0.093 | 0.103 |
|  | SD | 0.052 | 0.027 | 0.022 | 0.598 | 0.430 | 0.377 | 0.121 | 0.091 | 0.086 | 0.084 | 0.063 | 0.059 |
|  | Min | 0.004 | 0.004 | 0.004 | 0.194 | 0.194 | 0.194 | 0.070 | 0.069 | 0.070 | 0.028 | 0.028 | 0.030 |
|  | Max | 0.867 | 0.535 | 0.295 | 7.630 | 4.851 | 3.434 | 1.470 | 1.056 | 0.745 | 0.993 | 0.646 | 0.561 |
|  | 75th Percentile | 0.020 | 0.014 | 0.019 | 0.967 | 0.444 | 1.161 | 0.309 | 0.159 | 0.229 | 0.081 | 0.114 | 0.107 |
|  | 25th Percentile | 0.005 | 0.005 | 0.006 | 0.255 | 0.285 | 0.243 | 0.097 | 0.105 | 0.084 | 0.067 | 0.056 | 0.084 |
