# Supplementary material for: Arterioembolic Characteristics of Differentially Diluted CaHA-CMC Gels Within An Artificial Macrovascular Perfusion Model
Source: Aesthet Surg J. 2025 Feb 19;45(6):645–53. doi: 10.1093/asj/sjaf028 (PMC12209786; doi:10.1093/asj/sjaf028)
Supplement: sjaf028_Supplementary_Data [file sjaf028_Supplementary_Data.zip › Table S3.docx]

**Table S3. Particle morphology attributes for each mixture at different flow rates.**

| Mixture | | Circularity (0-1) | | | Aspect Ratio | | | Roundness | | | Solidity | | |
| --- | --- | --- | --- | --- | --- | --- | --- | --- | --- | --- | --- | --- | --- |
|  |  | Low Flow | Medium Flow | High Flow | Low Flow | Medium Flow | High Flow | Low Flow | Medium Flow | High Flow | Low Flow | Medium Flow | High Flow |
| Undiluted | Mean | 0.746 | 0.713 | 0.780 | 1.686 | 1.481 | 1.577 | 0.668 | 0.734 | 0.736 | 0.808 | 0.821 | 0.841 |
|  | SD | 0.253 | 0.256 | 0.240 | 0.684 | 0.521 | 1.411 | 0.201 | 0.181 | 0.189 | 0.104 | 0.096 | 0.088 |
|  | Min | 0.095 | 0.182 | 0.092 | 1.000 | 1.000 | 1.000 | 0.161 | 0.202 | 0.036 | 0.512 | 0.488 | 0.516 |
|  | Max | 1.000 | 1.000 | 1.000 | 6.226 | 4.941 | 27.599 | 1.000 | 1.000 | 1.000 | 1.000 | 1.000 | 1.000 |
|  | 75th Percentile | 0.958 | 0.465 | 0.870 | 1.015 | 1.256 | 1.366 | 0.985 | 0.796 | 0.732 | 0.941 | 0.691 | 0.897 |
|  | 25th Percentile | 0.650 | 0.734 | 1.000 | 3.012 | 1.497 | 1.020 | 0.332 | 0.668 | 0.981 | 0.741 | 0.766 | 0.933 |
| 1 to 0.5 | Mean | 0.726 | 0.667 | 0.749 | 1.593 | 1.652 | 1.612 | 0.693 | 0.665 | 0.706 | 0.812 | 0.802 | 0.844 |
|  | SD | 0.249 | 0.226 | 0.216 | 0.601 | 0.619 | 0.944 | 0.190 | 0.179 | 0.189 | 0.099 | 0.090 | 0.085 |
|  | Min | 0.180 | 0.172 | 0.061 | 1.000 | 1.000 | 1.000 | 0.176 | 0.112 | 0.069 | 0.430 | 0.469 | 0.260 |
|  | Max | 1.000 | 1.000 | 1.000 | 5.682 | 8.918 | 14.588 | 1.000 | 1.000 | 1.000 | 1.000 | 1.000 | 1.000 |
|  | 75th Percentile | 0.950 | 0.427 | 0.613 | 1.069 | 3.153 | 1.619 | 0.935 | 0.317 | 0.618 | 0.893 | 0.807 | 0.839 |
|  | 25th Percentile | 1.000 | 0.869 | 0.694 | 1.135 | 1.435 | 2.013 | 0.881 | 0.697 | 0.497 | 0.857 | 0.750 | 0.882 |
| 1 to 1 | Mean | 0.649 | 0.612 | 0.699 | 1.749 | 1.821 | 1.753 | 0.632 | 0.608 | 0.645 | 0.768 | 0.762 | 0.809 |
|  | SD | 0.250 | 0.231 | 0.217 | 0.623 | 0.696 | 1.052 | 0.183 | 0.173 | 0.187 | 0.109 | 0.104 | 0.093 |
|  | Min | 0.103 | 0.109 | 0.061 | 1.000 | 1.000 | 1.000 | 0.130 | 0.080 | 0.026 | 0.398 | 0.350 | 0.305 |
|  | Max | 1.000 | 1.000 | 1.000 | 7.715 | 12.548 | 37.924 | 1.000 | 1.000 | 1.000 | 1.000 | 1.000 | 1.000 |
|  | 75th Percentile | 0.635 | 0.316 | 0.276 | 1.698 | 1.785 | 2.453 | 0.589 | 0.560 | 0.408 | 0.822 | 0.696 | 0.526 |
|  | 25th Percentile | 1.000 | 0.555 | 1.000 | 1.372 | 2.666 | 1.295 | 0.729 | 0.375 | 0.772 | 0.957 | 0.750 | 0.857 |
| 1 to 2 | Mean | 0.651 | 0.664 | 0.657 | 1.694 | 1.878 | 1.803 | 0.645 | 0.593 | 0.608 | 0.755 | 0.754 | 0.755 |
|  | SD | 0.244 | 0.233 | 0.221 | 0.575 | 0.730 | 0.613 | 0.176 | 0.177 | 0.170 | 0.111 | 0.113 | 0.106 |
|  | Min | 0.074 | 0.102 | 0.103 | 1.000 | 1.000 | 1.000 | 0.134 | 0.049 | 0.097 | 0.199 | 0.369 | 0.298 |
|  | Max | 1.000 | 1.000 | 1.000 | 7.483 | 20.535 | 10.350 | 1.000 | 1.000 | 1.000 | 1.000 | 1.000 | 1.000 |
|  | 75th Percentile | 0.266 | 0.907 | 0.180 | 3.802 | 1.385 | 2.135 | 0.263 | 0.722 | 0.468 | 0.597 | 0.873 | 0.384 |
|  | 25th Percentile | 0.976 | 0.709 | 1.000 | 1.450 | 1.888 | 1.000 | 0.690 | 0.530 | 1.000 | 0.815 | 0.741 | 0.857 |
