# Supplementary material for: Arterioembolic Characteristics of Differentially Diluted CaHA-CMC Gels Within An Artificial Macrovascular Perfusion Model
Source: Aesthet Surg J. 2025 Feb 19;45(6):645–53. doi: 10.1093/asj/sjaf028 (PMC12209786; doi:10.1093/asj/sjaf028)
Supplement: sjaf028_Supplementary_Data [file sjaf028_Supplementary_Data.zip › Table S4.docx]

**Table S4. Summary of published CaHA-CMC product-associated ischemic adverse case reports and case series.**

| **Publication** | **Year** | **Treatment Indication** | **Complication(s)** | **Age** | **Gender** | **Product Dilution***  **(Product:Diluent)** |
| --- | --- | --- | --- | --- | --- | --- |
| Georgescu D et al.^24^ | 2009 | Glabellar Treatment | Skin Ischemia | 55 | M | Non-hyperdiluted |
|  |  | Nasolabial Fold Effacement | Skin Ischemia | 58 | F | Non-hyperdiluted |
| Sung MS et al.^25^ | 2010 | Nasal Augmentation | Skin, Ocular/Periorbital Ischemia | 25 | M | Non-hyperdiluted |
| Allen SH et al.^26^ | 2011 | Glabellar Treatment | Skin, Ocular/Periorbital Ischemia | 47 | F | Non-hyperdiluted |
| Dayan SH et al.^27^ | 2011 | Nasolabial Fold Effacement | Skin Ischemia | 39 | F | Undiluted |
|  |  | Nasolabial Fold Effacement | Skin Ischemia | 43 | F | Non-hyperdiluted |
|  |  | Nasolabial Fold Effacement | Skin Ischemia | 36 | F | Non-hyperdiluted |
| Beer K et al.^28^ | 2012 | Nasolabial Fold Effacement | Skin Ischemia | 40 | M | Hypodiluted (~1:0.1) |
|  |  | Nasolabial Fold Effacement | Skin Ischemia | 49 | M | Non-hyperdiluted |
| Kim YJ and Choi KS^29^ | 2013 | Nasal Augmentation | Ocular/Periorbital Ischemia | 30 | M | Non-hyperdiluted |
| Chang TY et al.^30^ | 2014 | Nasal Augmentation | Ocular/Periorbital Ischemia | 34 | F | Non-hyperdiluted |
| Darling MD et al.^31^ | 2014 | Cheek Volume | Skin Ischemia | 46 | M | Hypodiluted (1:0.2) |
| Hsiao SF and Huang HY^32^ | 2014 | Nasal Augmentation | Ocular/Periorbital Ischemia | 32 | F | Non-hyperdiluted |
| Tracy L et al.^33^ | 2014 | Nasolabial Fold Effacement | Skin Ischemia | 41 | F | Non-hyperdiluted |
| Chou CC et al.^34^ | 2015 | Nasal Augmentation | Skin, Ocular/Periorbital Ischemia | 35 | F | Non-hyperdiluted |
| Hsieh H et al.^35^ | 2015 | Nasal Augmentation | Skin, Ocular/Periorbital Ischemia | 47 | F | Non-hyperdiluted |
|  |  | Nasal Augmentation | Ocular/Periorbital Ischemia | 33 | F | Non-hyperdiluted |
| Cohen E et al.^36^ | 2016 | Nasal Augmentation | Skin, Ocular/Periorbital Ischemia | 24 | F | Non-hyperdiluted |
| Dominguez S et al.^37^ | 2017 | Nasolabial Fold Effacement | Skin, Ocular/Periorbital Ischemia | 38 | F | Non-hyperdiluted |
| Glass D et al.^38^ | 2017 | Temporal Augmentation | Ocular/Periorbital Ischemia | 64 | F | Non-hyperdiluted |
| Won SJ and Woo SH^39^ | 2017 | Vocal Cord Medialization | Pulmonary Embolism | 63 | F | Undiluted |
| Marumo Y et al.^40^ | 2018 | Nasal Augmentation | Skin, Ocular/Periorbital Ischemia | 26 | F | Non-hyperdiluted |
| Sung WI et al.^41^ | 2018 | Nasal Augmentation | Ocular/Periorbital Ischemia | 24 | F | Non-hyperdiluted |
| Vu PQ et al.^42^ | 2018 | Nasal Augmentation | Skin, Ocular/Periorbital Ischemia | 51 | F | Non-hyperdiluted |
| Oh DJ et al.^43^ | 2019 | Glabellar Treatment | Ocular/Periorbital Ischemia | 48 | F | Non-hyperdiluted |
| Uittenbogaard D et al.^44^ | 2019 | Temporal Augmentation | Skin Ischemia | 46 | F | Non-hyperdiluted |
| Liu YC et al.^45^ | 2020 | Nasal Augmentation | Skin, Ocular/Periorbital Ischemia | 25 | F | Non-hyperdiluted |
| Van Loghem J et al.^46^ | 2020 | Nasolabial Fold Effacement | Skin Ischemia | - | F | Non-hyperdiluted |
|  |  | Nasal Augmentation | Skin Ischemia | 34 | F | Undiluted |
| Mingazova L et al.^47^ | 2021 | Cheek Augmentation | Trigeminal Neuropathy | 39 | F | Non-hyperdiluted |
| Williams MN and Burgess C^48^ | 2021 | Cheek Volume | Skin Ischemia | 59 | M | Unspecified |
| Lindgren AL and Welsh KM^49^ | 2022 | Nasolabial Fold Effacement | Skin Ischemia | 28 | F | Non-hyperdiluted |
| Soares DJ and Blevins LW^50^ | 2022 | Cheek Volume | Skin Ischemia | 64 | F | Non-hyperdiluted |
| Virdi GS and Spotswood E^51^ | 2022 | Chin Augmentation | Skin Ischemia | 23 | F | Non-hyperdiluted |
| Hartman N et al.^52^ | 2023 | Cheek Volume | Skin Ischemia | 29 | F | Unspecified |
| Kadouch et al.^53^ | 2024 | Chin Augmentation | Skin Ischemia | - | - | Non-hyperdiluted |
|  |  | Cheek Volume | Skin Ischemia | 53 | F | Hyperdiluted (1:2) |

* Hypodilution - Aqueous diluent volume < 100% product volume; Hyperdilution - Aqueous diluent volume ≥ than 100% product volume.
