# Supplementary figures and images for: Arterioembolic Characteristics of Differentially Diluted CaHA-CMC Gels Within An Artificial Macrovascular Perfusion Model
Source: Aesthet Surg J. 2025 Feb 19;45(6):645–53. doi: 10.1093/asj/sjaf028 (PMC12209786; doi:10.1093/asj/sjaf028)

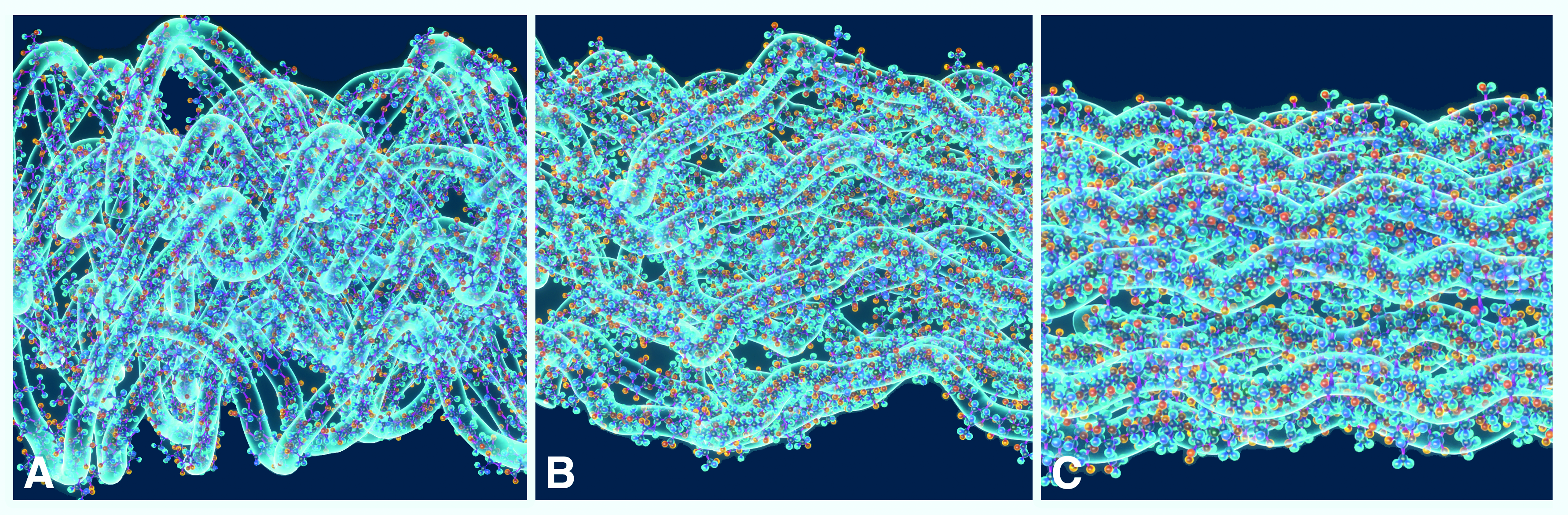

Supplement: sjaf028_Supplementary_Data [file sjaf028_Supplementary_Data.zip › Figure S1.tif]
